# On the equivalence of the X-ray scattering retrieval with beam tracking and analyser-based imaging using a synchrotron source

**DOI:** 10.1088/1361-6463/acee8c

**Published:** 2023-08-18

**Authors:** C Peiffer, L Brombal, C J Maughan Jones, F Arfelli, A Astolfo, D Dreossi, M Endrizzi, C K Hagen, A Mazzolani, R Menk, L Rigon, A Olivo, P R T Munro

**Affiliations:** 1 Department of Medical Physics and Biomedical Engineering, University College London, Gower Street, WC1E 6BT London, United Kingdom; 2 Department of Physics, University of Trieste, Via Valerio 2, 34127 Trieste, Italy; 3 Istituto Nazionale di Fisica Nucleare, Sezione di Trieste, Via Valerio 2, 34127 Trieste, Italy; 4 Elettra Sincrotrone Trieste SCpA, S. S. 14 km 163.5, 34012 Basovizza (TS), Italy; 5 Department of Computer and Electrical Engineering, Midsweden University, Sundsvall, Sweden

**Keywords:** beam tracking, analyzer based imaging, USAXS, x-ray dark-field, quantitative comparison

## Abstract

X-ray phase contrast imaging (XPCI) methods give access to contrast mechanisms that are based on the refractive properties of matter on top of the absorption coefficient in conventional x-ray imaging. Ultra small angle x-ray scattering (USAXS) is a phase contrast mechanism that arises due to multiple refraction events caused by physical features of a scale below the physical resolution of the used imaging system. USAXS contrast can therefore give insight into subresolution structural information, which is an ongoing research topic in the vast field of different XPCI techniques. In this study, we quantitatively compare the USAXS signal retrieved by the beam tracking XPCI technique with the gold standard of the analyzer based imaging XPCI technique using a synchrotron x-ray source. We find that, provided certain conditions are met, the two methods measure the same quantity.

## 1. Introduction

X-ray phase contrast imaging (XPCI) was first reported in 1965 when Bonse and Hart built the first single-crystal x-ray interferometer [1]. Since then, partly because of the availability of extremely brilliant and coherent synchrotron x-ray sources, many other realizations of XPCI have been developed, namely propagation-based imaging [2], crystal interferometry [1, 3], grating interferometry [4, 5] (GI), speckle-based imaging [6, 7], crystal-analyzer based imaging [8–10] (ABI), edge illumination [11] (EI) and beam tracking [12] (BT).

These techniques have in common that they make use of at least one complementary contrast mechanism, additional to conventional absorption contrast. Each technique exploits x-ray refraction as means of contrast, which arises due to differences in the real part of the refractive index of different materials. Most of these techniques can furthermore produce contrast based on ultra small angle x-ray scattering (USAXS). This scattering contrast reflects information about the subresolution microstructure of a sample and is a result of multiple refraction events [13].

The angular scattering distribution $g(\theta)$ of a sample describes how the sample scatters the incoming x-ray radiation $I_\mathrm{in}(\theta)$. The resulting local angular intensity distribution can therefore be modeled by the convolution [14, 15]

\begin{equation*} I(\theta) = I_\mathrm{in}(\theta)*g(\theta). \end{equation*}

For periodic structures the scattering distribution shows distinct peaks resembling the main lattice directions but for random microstructures (which is usually the case for the feature sizes in the USAXS regime) $g(\theta)$ can often be approximated by a Gaussian.

Indeed, it is possible to fully retrieve the local 1D scattering distribution in the USAXS regime [16–18], and even the full 2D scattering distribution [19], but this requires iterative deconvolution or, in the case of GI, the use of different wavelengths or propagation distances [18]. Instead, often a parametric description of the scattering distribution is used. The absorption, refraction and scattering -signals are then determined by the area under, the mean angle and the standard deviation of the scattering distribution, respectively [20, 21].

USAXS information is usually used for the structural analysis of materials on a scale bigger than 100 nm but smaller than the diffraction limit of optical microscopes. Materials investigated with USAXS include colloids [22, 23], polymers [24] and cement [25]. In XPCI the USAXS signal has been shown to be useful in various applications such as, for example, the better visualization [26, 27] and the quantification [28] of microbubbles used as contrast agents. Moreover, a dependence of the scattering signal on lung disease state was observed [17, 29, 30]. Recently, it has been reported that the scattering signal of EI, in combination with the absorption signal, and the dependence on the radiation energy can help distinguishing threat materials from benign materials in a security control [31].

BT is an alternative XPCI method, known to provide equivalent signals as EI [12]. It is capable of using incoherent polychromatic sources and, hence, is usable with compact lab sources [11, 32, 33]. Furthermore, it uses a very simple setup compared with most other XPCI techniques and is robust to mechanical vibrations. BT has the advantage over EI that there is no need for aligning two masks to each other and that it is possible to extract absorption, refraction and USAXS information from a single frame. Since ABI is a conceptually well established XPCI method with a long research history, and its working principle is related to BT, in this study, we investigate if the scattering signal obtained from a BT setup is equivalent to the scattering signal of the gold standard of ABI. This would unify historical research results from ABI and BT/EI into one body of research, and would allow researchers an alternative to using crystals for USAXS measurements. In this study, we compare the two setups using a partially, spatially coherent, monochromatic synchrotron x-ray source.

## 2. Imaging methods

**Figure 1.**
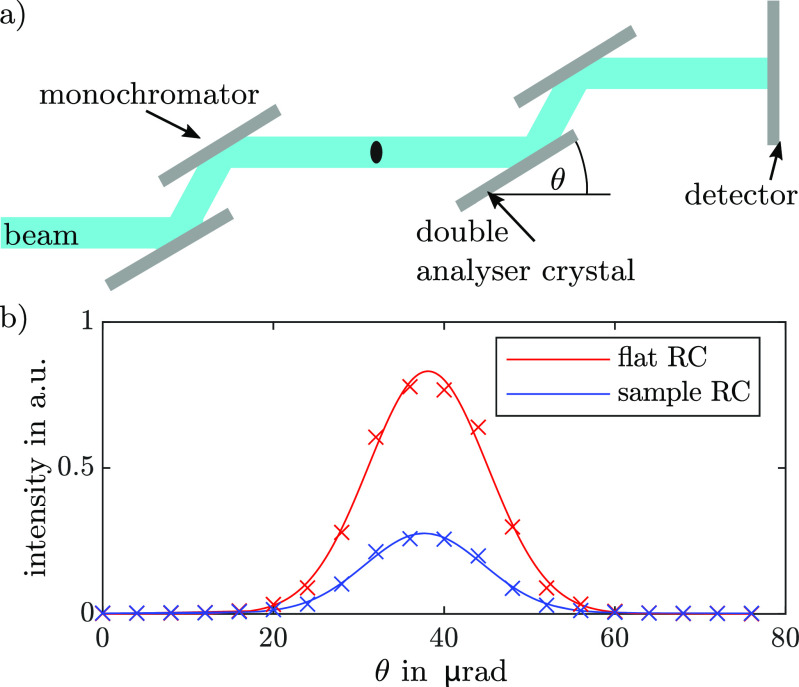
(a) Sketch of the ABI setup: The almost parallel polychromatic synchrotron beam is monochromatized by a double crystal. The monochromatic beam then interacts with the sample and the resulting scattering distribution is analyzed with a double crystal analyzer by rotating it and recording the intensity with respect to the rocking angle *θ*. (b) Gaussian fit of an experimental flat and sample RC. The introduced sample decreases the area under and changes the width and the mean angle of the RC. The FWHM of the flat RC is around 18.2 *µ*rad.

ABI is a coherent monochromatic XPCI technique that uses a perfect Si crystal, that has a very narrow reflectivity curve, to analyze the radiation scattered by a sample [10] (figure 1(a)). This is achieved by recording a so-called rocking curve (RC) which is depicted in figure 1(b), where the analyzer crystal is incrementally rotated, acting as an angular filter, and for each rocking angle and pixel the intensity is recorded. The flatfield RC for one pixel is then given by

\begin{equation*} \mathrm{RC}_\mathrm{flat}(\theta) = I_0(\theta)*R(\theta), \end{equation*}

where $I_0(\theta)$ is the angular x-ray beam distribution incident on the analyzer and $R(\theta)$ is the reflectivity curve of the analyzer crystal. Introducing a sample into the x-ray beam will alter the shape of this RC depending on the x-ray properties of the sample so that

\begin{equation*} \mathrm{RC}_\mathrm{sample}(\theta) = \mathrm{RC}_\mathrm{flat}(\theta)*g(\theta), \end{equation*}

where $g(\theta)$ is the scattering distribution of equation (1). Absorption will lead to a reduction of the area under the sample RC with respect to the flatfield RC, refraction will shift the mean angle and sub resolution scattering will change the width of the curve. Since in ABI the spatial resolution is determined by the pixel size, features that are smaller than the pixel size will refract the incident x-rays in all directions, usually randomly, and hence lead to a broadening of the RC instead of a shift of the mean angle of the RC [13].

In order to fully retrieve the scattering function $g(\theta)$, a deconvolution of the sample RC with the flatfield RC is necessary, which is lengthy and usually does not give a distinct result unless the RC is sampled very finely and the signal to noise ratio is very high [34]. Instead, often a random distribution of scatterers and hence a Gaussian scattering function is assumed. Then, the parameters of the curves can be obtained by fitting a Gaussian function to the RC [35, 36] or by using the much faster and easy to implement moment retrieval [14, 37], which gives direct access to the moments of the scattering function [21]. Alternative approaches that rely on three images only are possible, although with some limitations [38–40]. In this experiment the moment retrieval method was used and hence the scattering signal $s_{ABI}^2$ was obtained by calculating for each pixel

\begin{equation*} s_\mathrm{ABI}^2 = \mathrm{Var}(\mathrm{RC}_\mathrm{sample}(\theta))-\mathrm{Var}(\mathrm{RC}_\mathrm{flat}(\theta)), \end{equation*}

where Var$(f(x))$ denotes the variance of *f*(*x*).

**Figure 2.**
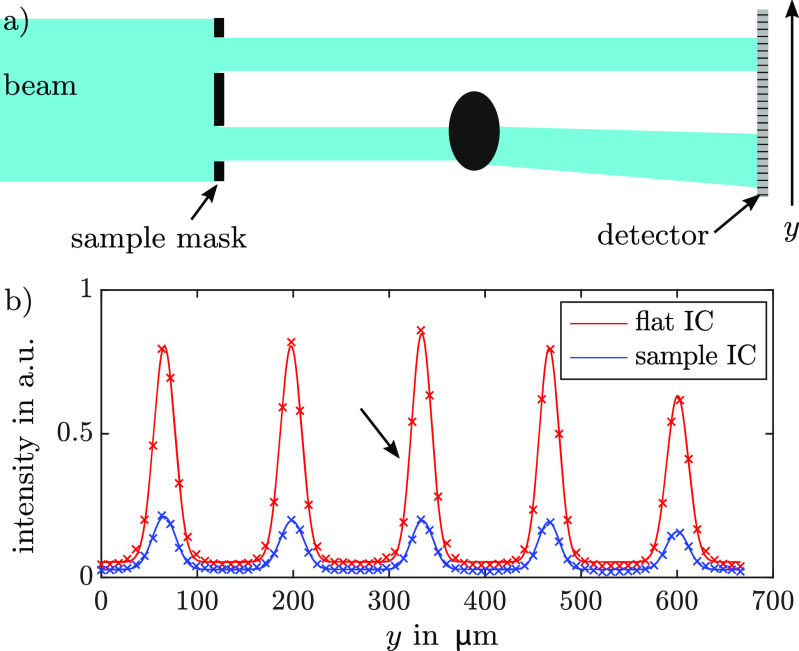
(a) Sketch of the BT setup: The monochromatic synchrotron beam is preshaped into beamlets by the sample mask. After interacting with a test object, the beamlet profiles are sampled via small (compared to the beamlet width) detector pixels. Note that the beamlet interacting with the sample changed its width and its mean direction. (b) Gaussian fit with offset of an experimental flat and sample IC for neighboring pixels. Note how the introduced sample changes the IC in a similar fashion to the RC in ABI. For comparison with the ABI setup: the FWHM of the flatfield beamlet marked with the arrow is around 17.5 *µ*rad.

EI and BT on the other hand, were originally also developed as a coherent monochromatic XPCI technique [41, 42], but because they do not need crystals and are not based on interference effects, they were readily translated to lab x-ray sources [11, 32, 33]. In BT, a so-called sample mask is placed immediately before the sample and divides the primary beam into small independent beamlets (see figure 2(a)). These beamlets then interact with the sample and are attenuated, refracted or broadened. Since the resolution in BT is determined by the aperture size of the sample mask, the change of the beamlet’s width originates from multiple refraction events caused by features smaller than the aperture width. In BT each beamlet’s intensity profile is sampled directly via detector pixels that are smaller than the beamlet’s width. This intensity profile is called illumination curve (IC) and is a concept similar to the RC in ABI. The flatfield IC for one pixel is therefore given by the convolution

\begin{equation*} \mathrm{IC}_\mathrm{flat}(y) = I(y)*M_\mathrm{sample}(y), \end{equation*}

where *I*(*y*) is the intensity profile of the x-ray beam and $M_\mathrm{sample}(y)$ is the transmission function of the sample mask. Introducing a sample into the beam will then alter the IC in a similar fashion as in ABI so that the sample IC for one pixel is given by

\begin{equation*} \mathrm{IC}_\mathrm{sample}(y) = \mathrm{IC}_\mathrm{flat}(y)*g(y), \end{equation*}

with $y = L\theta$ and *L* the distance from the sample to the detector. Figure 2(b) shows an experimental intensity profile with and without a sample for a few beamlets. Windows around each beamlet were cropped in order to allow the analysis of the resulting flatfield and sample IC in the same way as the RC by using Gaussian fitting [43] or moment retrieval [21]. Here, the moment retrieval was used and therefore the scattering signal $s_{BT}^2$ was obtained by calculating

\begin{equation*} s_\mathrm{BT}^2 = \mathrm{Var}(\mathrm{IC}_\mathrm{sample}(\theta))-\mathrm{Var}(\mathrm{IC}_\mathrm{flat}(\theta)). \end{equation*}

The result of this is a scattering image with a sampling step size in the phase sensitive direction (here vertical) equal to the mask pitch. In order to increase the sampling frequency, dithering can be applied which means that the sample is scanned through the beam in steps equal to whole fractions of the mask pitch and the separately retrieved projections are stitched together.

## 3. Experimental setup and materials

The experiment was performed at the SYRMEP beamline at the ELETTRA synchrotron in Trieste. The beam has the shape of a blade with a height of 4 mm and the effective source FWHM is 327 *µ*m in the horizontal and 66 *µ*m in the vertical phase sensitive direction. In this experiment the beam energy was set to 17.5 keV by using a double crystal Si (111) monochromator and the source to sample distance was 22.5 m. For ABI a double crystal analyzer was used with an angular step size of 4 *µ*rad. For a more thorough description of the ABI setup the reader is referred to Menk *et al* [44]. For the BT setup a mask obtained by laser ablation, which had a pitch of 122 *µ*m, was positioned immediately before the sample. The width of the apertures could not be precisely controlled using laser ablation and therefore ranges from 10 *µ*m to 15 *µ*m. In BT this is not an issue because the BT retrieval accounts for variations in beamlet sizes by using the flatfield IC for each beamlet as a reference. The detector was placed 140 cm downstream of the sample. The physical pixel size is 4.5 *µ*m but by binning a pixel size of 9 *µ*m was realized. In order to obtain a similar resolution to that of ABI we used 20 dithering steps, which, when accounting for magnification, correspond to a dithering step size of 5.65 *µ*m. Also, the sample to detector distance was chosen in order to obtain a flatfield IC that has a similar angular width as the RC of approximately 20 *µ*rad. The raw data of this study can be found in [45].

**Figure 3.**
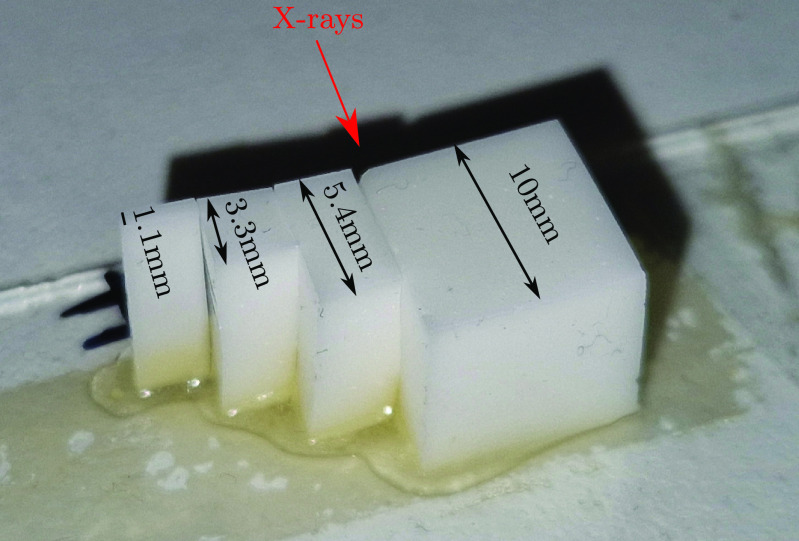
Exemplary Silicone step phantom with $\mathrm{TiO}_{2}$ scatterers. For each phantom the same dimensions were used.

In order to cover a broad range of scattering powers, we used phantoms made of Silicone (Elastosil RT 601 A/B, Wacker ChemieAG) and Gel Wax (Mindsets Online) as matrix materials with either $\mathrm{SiO}_{2}$ (monodisperse Silica particles, $\varnothing = 1\,\mu\mathrm{m}$, Pinfire—Gems and Colloids) or $\mathrm{TiO}_{2}$ (Product 224 227, Titanium(IV) oxide particles, $\varnothing\lt5\,\mu\mathrm{m}$, Sigma Aldrich) particles as scatterers. For each combination we were using phantoms of thicknesses 1.1 mm, 3.3 mm, 5.4 mm and 10 mm, respectively, in the beam direction (see figure 3) and different scatterer concentrations that are listed in table 1. For more information on the phantom making process the reader is referred to Jones *et al* [43, 46].

**Table 1.** Used mixtures of matrix material with scatterers.

Matrix + scatterer	scatterer concentrations in w%						
Silicone + $\mathrm{SiO}_{2}$	7.04	13.22	18.05	21.94	26.65		
Silicone + $\mathrm{TiO}_{2}$	0.11	0.18	0.32	0.43	0.53	0.69	1.25
Gel Wax + $\mathrm{SiO}_{2}$	6.82	12.78	17.99	22.73			
Gel Wax + $\mathrm{TiO}_{2}$	0.18	0.36	0.53	0.71	0.88		

## 4. Results and discussion

**Figure 4.**
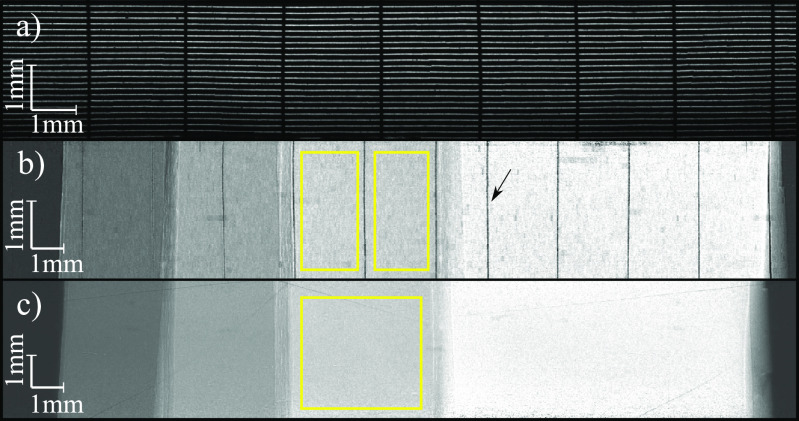
(a) x-ray projection image of the BT sample mask. The supporting bridges are visible as dark vertical lines. (b) Exemplary scattering image of a step phantom obtained by BT beam tracking. Due to absorption of the x-rays the supporting bridges produce artefacts (arrow). Hence, the ROI for a certain material (indicated in yellow) has to be divided. (c) scattering image of the same step phantom as in (b) obtained with ABI. Note, that the position of the sample is not the same as in the BT image so choosing the identical ROI for data processing is not possible.

Figure 4 shows how image artefacts (bridges in mask) and different sample positions rendered it impossible to choose the identical regions of interest for the comparison between ABI and BT. In order to calculate average values for a certain sample type, regions of interest (ROI) were manually defined so as to exclude these artefacts.

**Figure 5.**
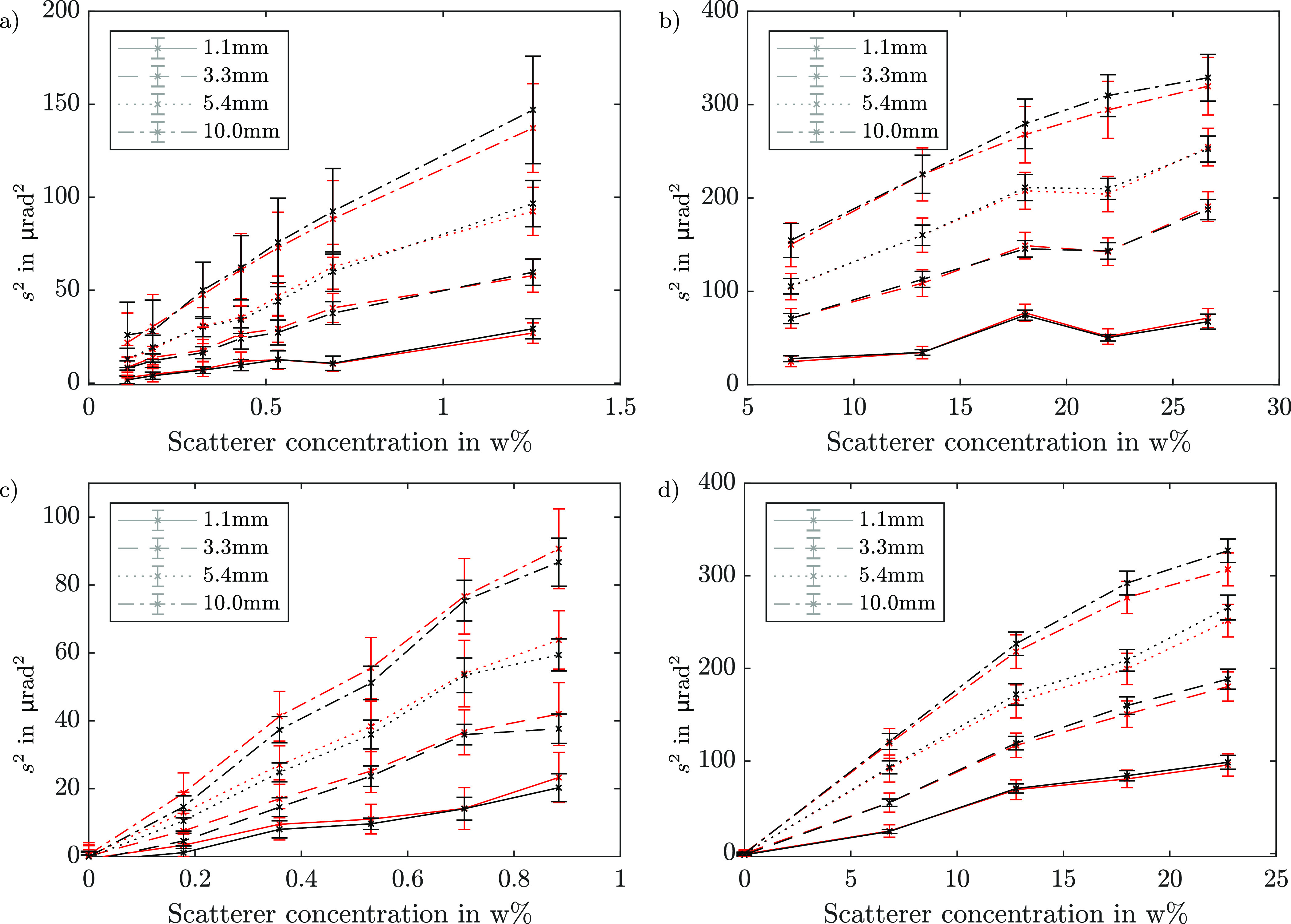
Comparison of the scattering signal obtained with BT (red crosses) and ABI (black crosses) for different matrix material/scatterer combinations and material thicknesses. The error bars resemble the standard deviation of *s* ^2^ in the respective ROI. (a) Silicone matrix with $\mathrm{TiO}_{2}$ micro particles. (b) Silicone matrix with $\mathrm{SiO}_{2}$ micro particles. (c) Gel wax matrix with $\mathrm{TiO}_{2}$ micro particles. (d) Gel wax matrix with $\mathrm{SiO}_{2}$ micro particles.

The result of this procedure can be seen in figure 5. The error bars are obtained by using the standard deviation of the chosen ROIs, and therefore also include the inhomogeneities in the spatial scatterer distribution. The average values for ABI and BT lie in each other’s uncertainty interval, which indicates a good match between the two techniques. Treating the whole BT and ABI datasets as two vectors, the correlation coefficient was calculated to be 0.9993 which indicates almost perfect direct proportionality. In order to statistically test if both methods actually measure the same quantity, we constructed a variant of the Kolmogorov-Smirnov (KS) test. Usually, this test is used in order to find out if two sample distributions stem from the same underlying probability distribution. This is done by comparing the absolute maximal difference, D, between the two sample cumulative distributions to a critical value for a certain significance level.

**Figure 6.**
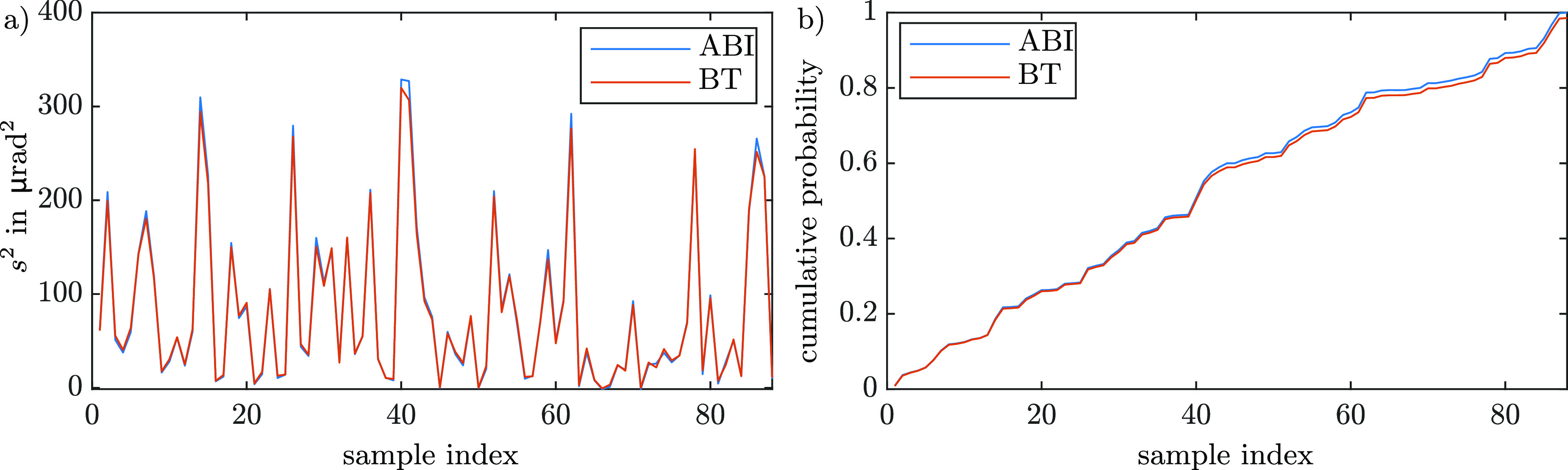
(a) Example plot of all the samples’ scattering values obtained from ABI and BT in a random order. (b) Cumulative distributions obtained from (a) by calculating the primitive of the two graphs reported in (a) and normalizing so that its integral is 1.

Figure 6 shows how we built the two sample cumulative distributions for this study. First, all the average scattering values for ABI and BT were plotted (figure 6(a)). We treated each of these two data sets as probability density distributions by normalizing to give a total probability of 1. Then, the cumulative probability distributions are obtained by calculating the indefinite integral of the two distributions (see figure 6(b)). The KS statistic D was finally calculated by

\begin{equation*} D = \mathrm{max}(abs(f_\mathrm{DEIcum}-f_\mathrm{EIcum})), \end{equation*}

where $f_\mathrm{DEIcum}$ and $f_\mathrm{EIcum}$ are the respective cumulative distributions of ABI and BT. Since changing the order of the samples while building the sample distribution changes D, D was calculated for 10^5^ different random orders of the samples. Each of these attempts can be seen as a test related to a different theoretical probability distribution. Accounting for the worst case, the highest D value was chosen to evaluate the test. For our dataset of 88 samples a maximal D value of 0.0204 was obtained. Since the critical value for a significance level of 5% and 88 samples is 0.205 and therefore much bigger than *D*, the null hypothesis that the two sample distributions, independent of how they were constructed, come from the same distribution, cannot be rejected.

We furthermore find that the uncertainty for BT is on average 3.35 *µ*rad (1.41 times) bigger than the uncertainty for ABI. The reason for this might be that in our experiment specifically the angular sampling step for ABI was 4 *µ*rad compared to a substantially bigger (due to experimental limitation) sampling step in BT of 6.4 *µ*rad. As a consequence, the RC in ABI was sampled at 20 positions with an exposure time of 1 s, which amounts to a total exposure time per retrieved pixel of 20 s. BT on the other hand is intrinsically a single shot approach, where only for the sake of increasing spatial sampling multiple exposures are needed. The exposure time for a single shot was 2 s and for the high resolution images we used 20 dithering steps so that the total imaging time was 40 s. The average total number of counts detected when sampling the flat field RC of one pixel was 7425 in the ABI case and 4545 for the IC in the BT case. The reader is referred to the supplementary material where we show the equivalence of both methods when using the same sampling step size and comparable photon statistics.

The visually good overlap between the BT and ABI data sets, the high correlation coefficient and the positive KS test let us conclude that indeed the technique of BT measures the same scattering quantity as ABI. A requirement for this is that the observed scatterers have dimensions smaller than the pixel size (ABI) or aperture size (BT). This paper compares ABI and BT using a synchrotron source because of the impracticalities of implementing ABI, a necessarily monochromatic method, with conventional sources. ABI, when using conventional sources, exploits only a very small portion of the source’s flux for example. Here, BT can extend ABI capabilities, since it can exploit the whole spectrum of conventional polychromatic sources. In the case of BT, the polychromaticity leads to an additional broadening of the beam, as described by Vittoria *et al* in [47]. It should also be noted that a comparison between scattering signals extracted with BT at synchrotron and conventional sources has been previously performed [48].

## Data Availability

The data that support the findings of this study are openly available at the following URL/DOI: https://doi.org/10.5522/04/22/787348.v1.
